# Thoracic surgical management of colorectal lung metastases: a questionnaire survey of members of the Society for Cardiothoracic Surgery in great Britain and Ireland

**DOI:** 10.1308/003588413X13511609956336

**Published:** 2013-03

**Authors:** S Jegatheeswaran, T Satyadas, AJ Sheen, T Treasure, AK Siriwardena

**Affiliations:** ^1^Central Manchester University Hospitals NHS Foundation Trust, UK; ^2^University College London, UK

**Keywords:** Colorectal cancer, Pulmonary metastases, Metastasectomy, Surgery

## Abstract

**Introduction:**

Distant metastases to liver and lung are not uncommon in colorectal cancer. Resection of metastases is accepted widely as the standard of care. However, there is no firm evidence base for this. This questionnaire survey was carried out to assess the current practice preferences of cardiothoracic surgeons in Great Britain and Ireland.

**Methods:**

An online questionnaire survey was emailed to cardiothoracic surgeons in Great Britain and Ireland. The survey was live for 12 weeks. Responses were collated with SurveyMonkey^®^.

**Results:**

Overall, there were 75 respondents. The majority (83%) indicated thoracic surgery as a specialist interest. Almost all (99%) used thoracic computed tomography (CT) for staging; 70% added liver CT and 51% added pelvic CT. Fluorodeoxyglucose positron emission tomography was used by 86%. The most frequent indication for pulmonary resection (97%) was solitary lung metastasis without extrathoracic disease. Video assisted thoracoscopic surgery (VATS) was used by 85%. In addition, thoracotomy was used by 96%. A third (33%) used radiofrequency ablation. Synchronous liver and lung resection was contraindicated for 83% of respondents. Over three-quarters (77%) thought that scientific equipoise exists presently for lung resection for colorectal lung metastases but only 21% supported a moratorium on this type of surgery until further evidence becomes available.

**Conclusions:**

The results confirm that the majority of respondents use conventional cross-sectional imaging and either VATS or formal thoracotomy for resection. The results emphasise the continuing need for formal randomised trials to provide evidence of any survival benefit from pulmonary metastasectomy for colorectal lung metastases.

Colorectal cancer is the third most common malignancy in the UK.^1^ Of patients with bowel cancer, a fifth have metastases at presentation and a quarter of patients who have resection of a primary colorectal cancer are subsequently found to have metastases.^2^ The most common sites of metastases are the liver and the lungs.^3^ Approximately 10–25% of patients with colorectal malignancy develop pulmonary metastases, of whom only 2.5% will have the metastases confined to the lungs alone.^4,5^ Pulmonary metastases are rarely symptomatic.

Hepatic resection has become accepted as the standard of care for patients with colorectal metastatic disease confined to the liver, and contemporary combination therapy with liver resection and chemotherapy is associated with longer survival compared with chemotherapy regimens without resection.^6,7^ The acceptance of liver resection without a randomised trial base comparing the treatment with chemotherapy alone has arguably produced a tendency to regard metastatic colorectal cancer as a surgically treatable disease rather than a systemic phenomenon. Studies from multi-institutional databases therefore report outcome from liver resection undertaken in the presence of extrahepatic disease and, in particular, in patients with lung metastatic disease.^8^

The management of patients with colorectal cancer lung metastases is multifaceted and is based on evidence short of randomised controlled trials. Colorectal cancer can metastasise to the liver by portal, nodal or direct spread. By the stage of pulmonary involvement, however, bowel cancer is a systemic disease.

Even in this setting, the continuance of technological advance has seen the advent of video assisted thoracoscopic resection of pulmonary metastases and the application of new non-resectional techniques such as radiofrequency ablation to treat metastases.^9,10^ In this regard, the current UK Pulmonary Metastasectomy in Colorectal Cancer (PulMiCC) trial promises to provide important evidence.^11^ However, pending completion and publication of this study, how are patients with colorectal lung metastases to be managed on a day-to-day basis? In order to obtain an overview of current practice, we undertook a questionnaire survey of cardiothoracic surgeons in Great Britain and Ireland.

## Methods

### Questionnaire design

An online questionnaire was prepared. This contained ten questions covering, in sequence, the area of practice of the respondent, tests used for staging and investigation of colorectal lung metastases, definition of current indications for pulmonary metastasectomy, methods for resection and/ or ablation, treatment in the setting of synchronous liver and lung disease, the position of the thoracic surgical consultation in the care pathway of a patient with pulmonary and liver metastases, views on synchronous liver and lung surgery, and views on current practice and future trial design in the absence of definitive randomised trial guidance. The full questionnaire can be seen in Appendix 1, which is available online only.

### Study population

The index population was the Great Britain and Ireland membership list of the Society for Cardiothoracic Surgery in Great Britain and Ireland (SCTS). No international members were contacted. This list is publicly available online (http://www.scts.org/). At the time of the study, the Great Britain and Ireland combined membership comprised 446 individuals. Seventy-nine did not have a valid email address and were excluded. Three members were deceased. Following all exclusions, electronic survey invitations were sent to 364 members with an online link to the questionnaire. The questionnaire went ‘live’ from 1 January 2011. At 6 weeks, a reminder was sent to the 246 members of the original 364 who were listed on the specialist register of the General Medical Council. The survey was closed to further responses after a total open period of 12 weeks. Overall, there were 76 respondents (21% of the original population of 364).

### Ethics committee approvals

The study was discussed with the trust’s research and development team, who advised that as there was no patient contact and only email contact with professional colleagues, no ethics committee submission was required.

### Data presentation

All categorical variables (yes/no responses) are presented as percentages. As the number of respondents to individual questions varied, the denominator for each question is the number of respondents to that particular question.

## Results

### Specialist interest of respondents

Of 75 respondents to this question, 62 (83%) indicated general thoracic surgery as their specialist interest. Thirty-eight (51%) also listed adult cardiac surgery as their specialist interest.

### Investigations in a patient referred for surgical resection of a solitary lung metastasis from colorectal cancer

**Figure 1:**
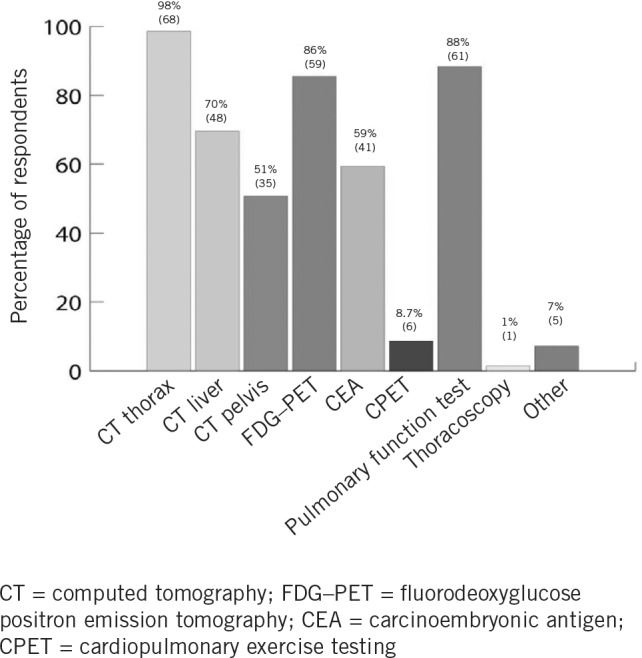
Investigations in a patient referred for surgical resection of a solitary lung metastasis from colorectal cancer

Of 69 respondents to this question, 68 (99%) used computed tomography (CT) of the thorax as a staging investigation while 48 (70%) added CT of the liver and 35 (51%) added CT of the pelvis. Fluorodeoxyglucose positron emission tomography (FDG–PET) was used by 59 surgeons (86%) while carcinoembryonic antigen was measured by 41 (59%). In terms of pre-operative fitness for surgery, 6 (9%) undertook cardiopulmonary exercise testing and 61 (88%) undertook pulmonary function tests. Thoracoscopy was undertaken by one surgeon (1%). Other investigations included chest radiography, mediastinoscopy, endobronchial ultrasonography, intra-operative flexible bronchoscopy and transoesophageal echocardiography. The distribution of responses to this question is shown in Figure 1.

### Current indications for thoracic surgical resection of lung metastases

**Figure 2:**
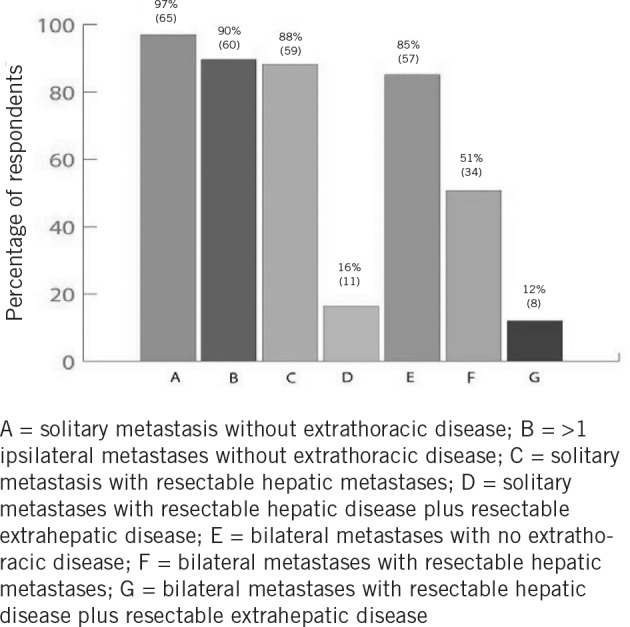
Indications for thoracic surgical resection of colorectal lung metastases

There were 67 replies to this question and the most frequent indication (*n*=65, 97%) was that of a solitary lung metastasis in the setting of no extrathoracic disease. A number of scenarios were offered including bilateral lung disease and extrapulmonary disease. The distribution of responses to this question is shown in Figure 2.

### Method of resection and/or ablation

Of 66 respondents to this question, 56 (85%) stated they would use video assisted thoracoscopic surgery (VATS). Thoracotomy and resection was also used by 63 surgeons (96%) while 22 (33%) used radiofrequency ablation.

**Is the presence of lung and liver metastatic disease in colorectal cancer a contraindication to surgery?**

Of 65 respondents to this question, 48 (74%) replied ‘no’.

**If liver resection is planned in the presence of lung metastasis, should the patient be reviewed by a thoracic surgeon prior to hepatectomy?**

Of 66 respondents to this question, 58 (88%) replied ‘yes’.

**If liver resection is planned in the presence of lung metastasis, can synchronous surgery be offered?**

Of 66 respondents to this question, 55 (83%) replied ‘no’.

**Are we still at a point of scientific equipoise as regards lung resection for colorectal lung metastases?**

Of 68 respondents to this question, 52 (77%) replied ‘yes’.

**should there be a moratorium on lung resection for colorectal pulmonary metastases until we see the results of a randomised controlled trial?**

Of 67 respondents to this question, 53 (79%) replied ‘no’. When asked a supplemental question as to whether the surgeon would participate in a UK-wide study to address this question, 35/41 respondents (86%) replied ‘yes’.

## Discussion

This questionnaire survey has focused on the thoracic surgical management of patients with lung metastases from colorectal cancer. The first issue to be considered when interpreting the results of online questionnaires is whether the appropriate target population was identified. In our opinion, accessing the UK and Ireland membership list of the SCTS can be regarded as a reasonable method for identifying the correct study population.

The second issue is the response rate. It should be borne in mind that this was a voluntary questionnaire sent by email with no incentives offered for completion. The SCTS has 45 consultant members who are exclusively thoracic (non-cardiac) surgeons and 60 whose practice includes some thoracic surgery. In this context, 76 respondents is an encouraging response. It is an important facet of the interpretation of questionnaires that undue extrapolation is avoided but we believe that this is representative of the views of the UK’s thoracic surgeons. There remains the possibility of bias. For example, it is possible that there was a preferential response from surgeons who favour thoracic resection for colorectal lung metastases. Having accepted these limitations, the population of 76 respondents and the replies to 10 selected questions comprise a comprehensive overview of contemporary thoracic surgical practice.

Looking in detail at the responses, it was to be expected that thoracic CT would feature as the mainstay of thoracic imaging (Fig 1). It was perhaps surprising for a survey undertaken in 2011 that FDG–PET was not more widely used and also, considering that many of these patients would already have undergone colonic and liver surgery, that more sophisticated tests of pulmonary reserve were not employed more frequently.

The question on current indications for pulmonary metastasectomy could possibly be regarded as leading because the options for response were fixed in the questionnaire (Fig 2). If this potential limitation is accepted, the reply to this question was perhaps the most insightful: even if one believes that respondents had a bias towards pulmonary resection, it seems that there is no clear consensus on the boundaries for resection. Bilateral lung disease and liver metastatic disease were considered an indication for resection by eight respondents to that question (12%).

In terms of method used for resection, most surgeons were prepared to use VATS and it was evident that either formal thoracotomy or VATS are the most popular. About a third of the respondents include radiofrequency ablation in their treatment plan.

The final questions sought to delve into the clinical paradigms involved in the management of patients with pulmonary metastatic disease from colorectal cancer. The majority of respondents stated that the presence of lung and liver metastatic disease was not in itself a contraindication to surgery. However, the uncertainty of respondents was reflected clearly in the mainly affirmative response to whether a position of scientific equipoise still existed in relation to the management of pulmonary metastases. The desire of clinicians to treat their patients, often in the setting of a lack of a substantial evidence base for invasive procedures, is highlighted in the lack of support for a moratorium on lung resection pending the results of any future trial.

## Conclusions

This 2011 study reports the results of an online questionnaire survey of cardiothoracic surgeons undertaking pulmonary resection for colorectal lung metastasis in the UK and Ireland. The study population was relatively small and the response should not be regarded as a formal position statement or evidence base.

The results confirm that the majority of respondents use conventional cross-sectional imaging and either VATS or formal thoracotomy for resection. The replies to specific questions on management shed interesting light on the lack of clear consensus on management: the majority of respondents stated that a position of equipoise exists in relation to lung resection for pulmonary colorectal metastases but equally that there should not be a moratorium pending any future availability of evidence. The results emphasise the continuing need for formal randomised trials in line with the Growing Recruitment in Interventional and Surgical Trials initiative, supported by an National Institute for Health Research Clinical Research Network working group.^12^ The need for better evidence concerning any survival benefit from pulmonary metastasectomy for colorectal lung metastases is a case in point.
